# Gaze-Assisted User Intention Prediction for Initial Delay Reduction in Web Video Access

**DOI:** 10.3390/s150614679

**Published:** 2015-06-19

**Authors:** Seungyup Lee, Juwan Yoo, Gunhee Han

**Affiliations:** 1 School of Integrated Technology, Yonsei University, Incheon 406-840, Korea; E-Mails: youb007@yonsei.ac.kr (S.L.); j.y@yonsei.ac.kr (J.Y.); 2 Yonsei Institute of Convergence Technology, Yonsei University, Incheon 406-840, Korea

**Keywords:** gaze assisted, user intention prediction, threaded interaction model, initial delay reduction, web video prefetching

## Abstract

Despite the remarkable improvement of hardware and network technology, the inevitable delay from a user's command action to a system response is still one of the most crucial influence factors in user experiences (UXs). Especially for a web video service, an initial delay from click action to video start has significant influences on the quality of experience (QoE). The initial delay of a system can be minimized by preparing execution based on predicted user's intention prior to actual command action. The introduction of the sequential and concurrent flow of resources in human cognition and behavior can significantly improve the accuracy and preparation time for intention prediction. This paper introduces a threaded interaction model and applies it to user intention prediction for initial delay reduction in web video access. The proposed technique consists of a candidate selection module, a decision module and a preparation module that prefetches and preloads the web video data before a user's click action. The candidate selection module selects candidates in the web page using proximity calculation around a cursor. Meanwhile, the decision module computes the possibility of actual click action based on the cursor-gaze relationship. The preparation activates the prefetching for the selected candidates when the click possibility exceeds a certain limit in the decision module. Experimental results show a 92% hit-ratio, 0.5-s initial delay on average and 1.5-s worst initial delay, which is much less than a user's tolerable limit in web video access, demonstrating significant improvement of accuracy and advance time in intention prediction by introducing the proposed threaded interaction model.

## 1. Introduction

In spite of remarkable hardware and network speed improvement, unavoidable delay from a user input action to a system response still exists, and it causes significant adverse effects on the system usability and user experiences (UXs). The initial delay in web video access is one of the most common inconveniences experienced in everyday life [1]. It has been reported that a 0.1-s delay is considered to be instantaneous access by the user, and a 2-s delay is the limit of user's patience for web page loading [2,3]. Most users tend to leave the web page when the initial delay of a web video exceeds 2 s, and only 2% of the users revisit the same site [1]. Web video prefetching based on user intention prediction is one of the attractive solutions to overcome the physical speed limits in the web environment.

The intention prediction has been studied and employed in various applications and services. The conventional user intention prediction techniques are categorized into a context-based intention prediction and an action-based intention prediction. The context-based intention prediction refers to a prediction scheme that prepares or executes a certain action before a user's request based on the user's patterns and preferences that have been accumulated from past usages. The predictive recommendation services in user contexts [4], auto-complete [5] for search engine query typing, text message prediction for quick typing [6] and the channel reordering [7] for Internet Protocol Television are examples of context-based prediction. In particular, web page prefetching for latency reduction can be implemented by predicting the probability of visiting the link based on past log data [8–10] using the Document Object Model (DOM) [11]. However, these techniques are futile for web video services, because the same video is rarely revisited, unlike other types of web services, and newly uploaded video contents cannot be analyzed with the previous DOM.

The action-based intention prediction refers to a prediction scheme based on users' action that can be sensed prior to the users' actual command action on the system. Auto-scroll by gaze tracking [12,13] and auto-wake-up for wearable devices [14] are examples of action-based prediction. Action-based intention prediction using a mouse have been intensively studied, because the mouse is the most typical and widely-used input device for computing. Various target prediction schemes based on movement vector analysis [15], the neural network algorithm [16], the Kalman filter algorithm [16,17], the kinematic template matching algorithm [18] and area cursor techniques [19–22] have been proposed to improve the target selection task. However, they are not suitable for current web applications due to the complicated layout of hyperlinks and the excessive computational consumptions. Furthermore, web data prefetching based on the cursor's dwell time, velocity [23] and moving direction [24,25] was proposed to reduce the web access latency, but it often results in false prefetching due to the user's meaningless cursor movement.

Meanwhile, a gaze tracker is widely used as an input device for various information devices [26–30] and even for a vehicle [31]. The gaze-only input technique often suffers from the so-called “Midas touch” problem [32]. This problem can be relieved by recognizing the intention based on in-depth processing of gaze tracking data, such as the intended and unintended gaze movement classification [33], or by introducing an additional modality for confirmation, such as hand gesture [34], touch action [35–37], joystick [38] and EEG (electroencephalography) [39]. As these previous studies aimed to utilize the gaze as an input method, it is more important to recognize the user intention accurately after the actual command action than before the command action. Besides the use of gaze as an input, the gaze can be used to infer the cognitive states and the contextual circumstance of the user in cognition-aware computing [40], such as a particular activity recognition [41,42], contextual cues recognition [43] and search target prediction [44].

These gaze-only intention prediction techniques are very advantageous to identify the context, propensity and preference of the user. However, they are not suitable for the web video prefetching application, because the early studies focused on the contextual and implicit intention about the user activity rather than the behavioral and explicit intention to click a target among chosen candidates. The implicit intention can be predicted by collecting large amounts of data and analyzing the collected data for a long period, while the behavioral intention requires the instant user behavior data for a short interval. Furthermore, the additional crucial parameters, such as distinctive characteristics of the web, network bandwidth consumption and the advance time of prediction before a user's actual command action, should be considered more, as well as the prediction accuracy for the web video prefetching application.

This paper has three major contributions. First, a threaded interaction model (TIM) is proposed to utilize the sequential and concurrent flow of multiple behavioral resources as a reflection of the cognitive resources. The second contribution is the composition of prediction system modules, which employs the cursor movement as a candidate selection module and the cursor-gaze relationship as a decision module, from the behavioral analysis of the web video application. The third contribution is the identification of the system design parameters and their influences on the web video access delay reduction, as well as the user perception based on the TIM.

The remainder of this paper is outlined as follows: Section 2 describes the proposed gaze-assisted user intention prediction with the TIM and prefetching scheme. Section 3 presents the details of the implementation system. Section 4 describes the experimental design, such as the design parameters, the performance measures and the test environment used. Section 5 provides the experimental results and the summarized performance of the proposed user intention prediction systems. Section 6 concludes with the implication of this paper.

## 2. The Proposed Gaze-Assisted User Intention Prediction

**Figure 1:**
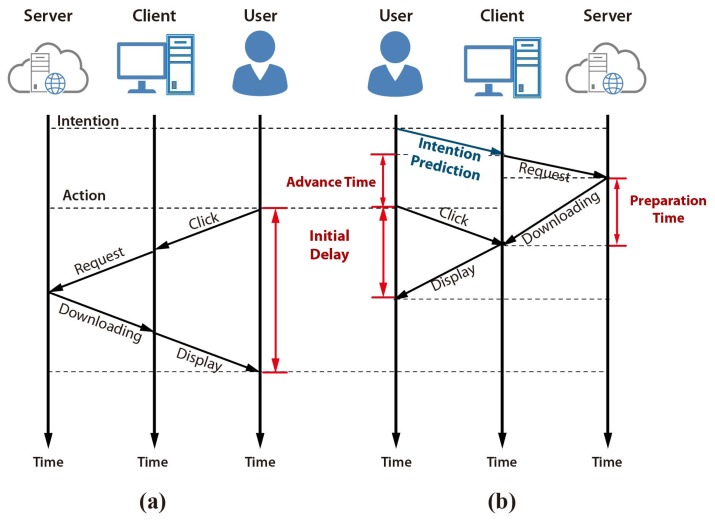
Process flow of web access. (**a**) Typical web video access; (**b**) web video access using the proposed user intention prediction.

Figure 1 shows how a user intention prediction can improve the initial delay in the web video access application using prefetching. A typical web video access process initiates the player first, and then downloads the streaming data after the input action, as shown in Figure 1a. The initial delay can be minimized by adapting prefetching based on a user intention prediction. Web access prefetching and video data preloading can be initiated before the actual input action if the user's intention can be predicted prior to the click action, as shown in Figure 1b. The effectiveness of prefetching is determined by the accuracy and the advance-time of the prediction.

### 2.1. Threaded Interaction Model

**Figure 2:**
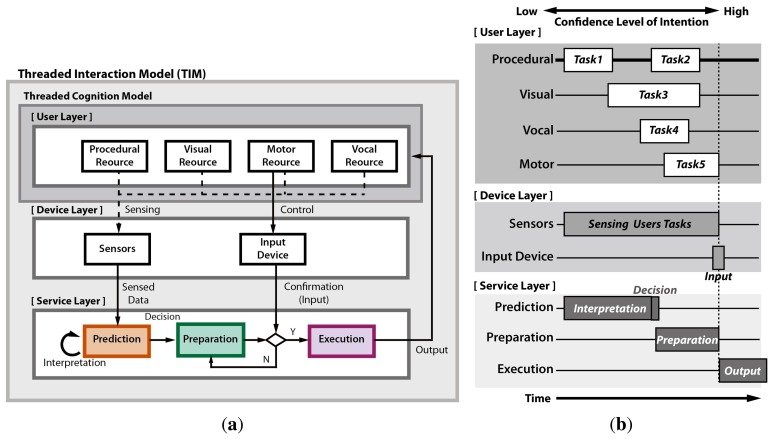
(**a**) General framework of threaded interaction model (TIM); (**b**) proposed TIM.

It is crucial to reflect the flow of thought in the user intention prediction scheme. We propose a threaded interaction model (TIM) based on a threaded cognition model [45,46], as shown in Figure 2. The TIM formulates an interaction framework that reflects the sequential and concurrent process flow of the interaction devices and service, as well as the human cognition as a thread. The TIM enables identifying the types of input devices, design parameters and decision criteria to activate the preparation proactively prior to actual input.

Figure 2a shows the general framework of the TIM. The TIM mainly consists of three layers: (1) a user layer; (2) a device layer; and (3) a service layer. Each layer contains multiple constructs. Figure 2b shows the sequential and concurrent flow of the multiple threads in each construct and layer along with the timeline. When a user uses a prediction system, the user's threads of each construct in the user layer are recognized by the sensors in the device layer. Then, the sensed data are interpreted to predict the user's intention as a pre-cue in the prediction construct. It is natural that the confidence level of intention gets higher as the time gets closer to the actual input action. Once the confidence level of intention exceeds a certain limit, *i.e.*, the collected pre-cue sufficiently reflects the intention, the prediction construct decides the activation of preparation. After the user makes the confirmation with the actual input, the execution construct provides the prepared actual response to the user.

**Figure 3:**
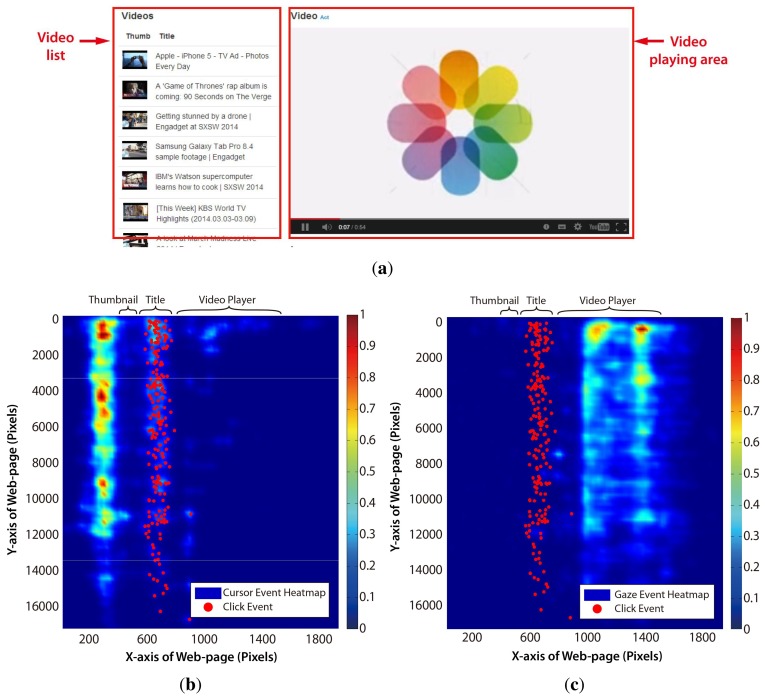
(**a**) A video website layout; (**b**) mouse event pattern of the video website used; (**c**) gaze event pattern of the video website used.

The TIM can be applied to develop the user intention prediction scheme suitable for web video access. First of all, as the visual (gaze actions) and manual (mouse actions) constructs are the most typical cognitive resources for web access in the desktop environment, the threads in these constructs should be carefully examined based on the TIM. The gaze and cursor movement pattern was obtained from 10 participants, who used a typical video website (see Figure 3a), accessing any video of their own interests. Unlike the common web page, the video website typically has two main graphical user interface (GUI) components, the video player region and the video list region, as shown in Figure 3a. Figure 3b and Figure 3c show that distinct user behavior was observed in this web page layout. Table 1 summarizes the horizontal distribution of cursor, gaze and click events in each area. The cursor hovered over the blank area around the video thumbnail image region for most of time, because the cursor was in a static state while watching the video and placed so as not to block the thumbnail images. Meanwhile, the gaze dwelled almost on the video player region. Saccadic gaze movements occasionally occurred when the users wanted to search other videos. The cursor transited to the dynamic state for click action when the user wanted to watch other videos.

This means that the cursor position or the gaze position itself does not represent the user's intended target. Furthermore, either the cursor or the gaze movement analysis cannot promise a sufficiently high confidence of intention. As the video player region performed as a “gaze trap” that gathered most of the users' attention, analyzing only the gaze movement pattern to find out the transition point between the saccadic and fixation movement is inappropriate in the web video application. Even if the fixation data in the video player were filtered, only depending on the gaze movement may cause the wrong intention prediction due to the “Midas touch” problem when the cursor is in the static state. Therefore, it is required to figure out the relationship between the gaze and the cursor behavior and to specify the multiple threads based on the TIM before the actual click action.

**Figure 4:**
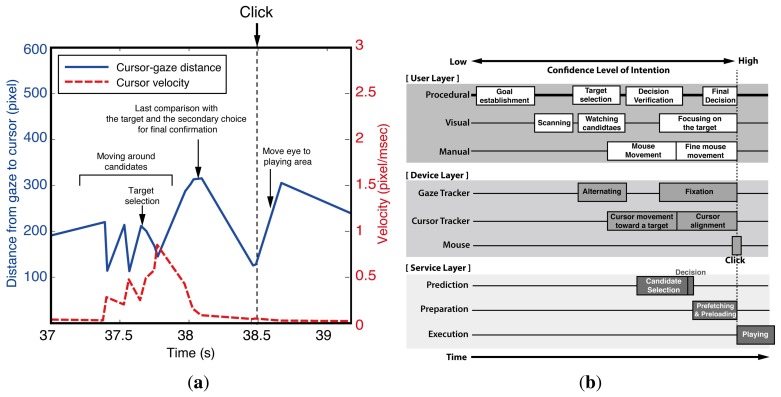
(**a**) An example of the cursor and gaze behavior; (**b**) the proposed threaded interaction model for user intention prediction in web video access.

Figure 4a briefly shows the observed cursor-gaze distance and the velocity of the cursor around a click event. The cursor-gaze distance and the cursor velocity repeatedly go up and down in opposite directions while the user meanders around the target candidates. Once the target is chosen, the user starts to move the mouse toward the target. Eventually, the gaze is fixed to the final target and tries to align the mouse toward the target, reducing the mouse speed. When the cursor reaches close enough to the target, the velocity of the mouse decreases, and the cursor-gaze distance decreases. It is worthy noting that many users tend to move their gaze again before the click action. This can be interpreted as a final comparison between the target and the secondary choice for the confirmation of the decision. After clicking the target, the cursor-gaze distance increases while the cursor is in the static state, because the user returns the gaze to the video player to watch the clicked video. This observed interaction between the visual and manual resources provides the information for how the prediction, preparation and execution thread should be aligned in the timeline and which task should be provided for each thread.

Figure 4b shows the TIM that is applied to the user intention prediction in web video access considering the above-mentioned user behavior. The process starts from a user's goal establishment, choosing the video content of interest, such as sports, and then, the user scans the web page to find links that are appropriate to the goal. The prediction starts when the cursor is moving toward the target to avoid the “Midas touch” problem. When the confidence level is sufficiently high, the decision for the prefetching (preparation) is made for the selected candidates. Then, the preparation thread in the service layer preloads the web video data to the local device waiting for the actual click action. If the user clicks the prepared link, the system immediately starts displaying the preloaded video. We can infer that the mouse and gaze tracker were the most appropriate input devices for intention prediction in the web video prefetching. Unlike the intuitive reasoning, the gaze position is not suitable for candidate selection, because it stays in the video player area most of the time. Rather, the cursor position is a better choice for candidate selection, because it stays around the video list area most of the time. The confidence level of intention to click one of the candidates drastically increases when the cursor-gaze distance decreases as the gaze moves toward the target to click before the actual click action. The cursor location serves as a pre-cue for the candidate selection, and the cursor-gaze distance serves as a pre-cue for the moment of click action.

## 3. Implementation

**Figure 5:**
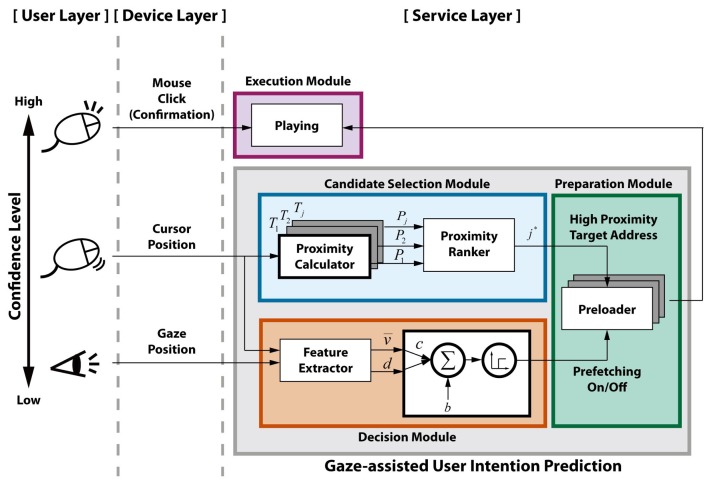
The proposed gaze-assisted user intention prediction based on TIM.

The proposed user intention prediction consists of a candidate selection module, a decision module and a preparation module based on the TIM, as shown in Figure 5. The candidate selection module selects prefetching target candidates based on the weighted proximity from the cursor to the target. The cursor position is chosen as an input for the candidate selection module considering the confidence level in user intention. Despite the fact that the gaze precedes the cursor, the saccadic eye movement causes too many target selections. The decision module computes the click possibility in the near future based on the cursor and gaze movement relationship and performs the actual prefetching when the click probability is sufficiently high. The high proximity target among the selected candidates starts to be preloaded firstly when prefetching is turned on by the decision module. The preparation module waits for the actual click action while preloading the filtered web video data as a buffer before the execution. When the user clicks the preloaded target properly, the execution module just plays that video.

The proximity (*P_j_*) between the cursor and the *j*-th target link (*T_j_*) on a web page is obtained from the overlapping area (*A_j_*) of a virtual circle, *C*, around the cursor with a radius of *R* and the target, *T_j_*. The proximity for target *T_j_* is calculated as follows:

(1) $$P_{j}=\int_{A_{j}}w\left(r\right)$$

where *w*(*r*) is a proximity weight function that is monotonically decreasing with respect to distance *r* from the cursor. As the distance, *r*, becomes larger, prefetching can be activated earlier, but the prediction accuracy decreases. The proximity weight function can be useful to provide different priorities when multiple targets that have different shapes from each other, such as a star-shape, a square, *etc.*, have the same proximity value. After the proximity calculator computes the proximity (*P_j_*) for each overlapping target, the proximity ranker chooses candidates that have a high proximity value. Then, the link addresses of the chosen targets are passed to the preloader (preparation module). Each preloader in the preparation module starts prefetching and preloading the given addresses when the decision module determines the click possibility.

**Figure 6:**
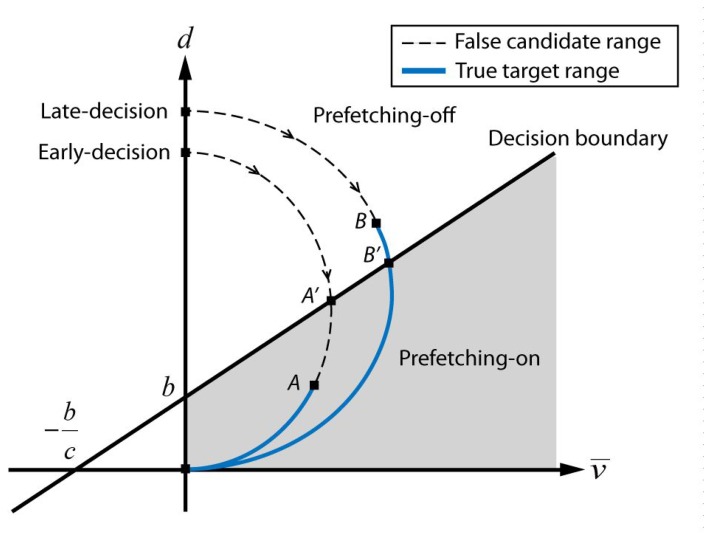
Typical target selections and decision boundary on the feature space.

Considering user behavior, the decision module computes the click possibility in the near future according to the low-pass filtered velocity of the cursor (*v̅*), and the distance between the cursor and the gaze position (*d*) as follows:

(2) $$O=f\;\left(d+c\bar{v}+b\right)$$

where *O* is the binary output to turn on the prefetching, *f* is a step function and c and *b* are coefficients associated with the decision boundary on the *v̅-d* feature space, as shown in Figure 6. Constant *c* determines the relative sensitivity of the cursor and the gaze motion. Constant *b* determines the area of the prefetching-on region. As the cursor velocity and the cursor gaze distance are positive, the prefetching-on region is placed in the first quadrant under the decision boundary given in Equation (2). If the prefetching-on region is excessively large, then meaningless prefetching for false candidates causes a waste of network bandwidth. On the other hand, if the prefetching-on region is too small, the prefetching is turned on only when the user is about to click the target. This late decision results in an insufficient preparation (preloading) time to reduce the initial delay. Therefore, the appropriate prefetching-on region adjustment is critical to optimize the trade-offs between the accuracy and advance time.

The cursor velocity and cursor-gaze distance change of typical target selection cases are illustrated on the feature space, as shown in Figure 6. After the target searching process, once the user decides a target to click fixing the gaze to the target, the user starts to move the cursor, which is originally placed in the static state with distance *d* from the target. During the approaching process, the cursor velocity starts to increase while the distance decreases, because the gaze is fixed to the target. When the cursor gets close enough to the target, the cursor velocity decreases and eventually stops on the target. Meanwhile, the candidate selection module generates candidates with the proximity boundary around the cursor while the cursor is heading toward the target. The actual prefetching is activated within the prefetching-on region regardless of whether it is a true target or not. For example, a curve, denoted as “early-decision” in Figure 6, passes through the decision boundary at the moment denoted by *A′*, and the proximity boundary around the cursor meets the true target at the moment denoted by *A*. The preparation module performs prefetching for all candidates that meet the virtual circle around the cursor when the curve passes *A* on the decision boundary. There exists the possibility to prefetch false targets during the *A′*−*A* period, thereby wasting network resources. In contrast, the second case, denoted as “late-decision” in Figure 6, shows that the proximity boundary around the cursor meets the true target at the moment denoted by *B* and passes the decision boundary at the moment denoted by *B′*. The preparation module starts prefetching after passing *B′* on the decision boundary. Hence, the *B*–*B′* range is the missed time period that can be utilized for preloading. This observation suggests that the choice for the decision boundary is the key design factor for minimizing the initial delay without wasting network resources.

Even though the proximity boundary (*R*) and the decision boundary parameters (*c* and *b*) in this paper were empirically determined from an intuitive analysis of the pre-collected behavior data for the simplicity of implementation, various adaptation schemes may be applicable to achieve a better performance by optimizing the parameters to reflect the user's specific behavior. However, even if the proximity boundary (*R*) can be adapted according to the cursor velocity and the moving direction, the prefetching performance is almost independent of the proximity boundary.

## 4. Experimental Design

### 4.1. Parameters

The choice of appropriate design parameters is a critical issue to optimize the system performance. The design parameters of the proposed gaze-assisted user intention prediction for web video access were carefully chosen according to the TIM and the Internet access process as follows.

The hit-ratio is the measure that represents whether any of the prefetched targets are clicked by the user. If the user clicks any link that has not been prefetched, then this means that the prediction has failed. Even though a high hit-ratio is obtained by either increasing the number of preparation modules or increasing the prefetching-on region, it may also increase false prefetching, which the wasting of network resources.

The preparation time indicates the time period from the start of the prefetching to the click action, *i.e.*, a preloading duration. A longer preparation time is obtained if the prefetching decision is made at the early intention stage by increasing the prefetching-on region or increasing the proximity boundary, *R*. A longer preparation time provides sufficient time to load the video player and the video stream at the cost of low prediction accuracy.

The downlink bandwidth is the measure of the downloading network speed per preloader during the preloading. The downlink bandwidth tends to decrease as the number of preparation modules increases, because the overhead for multiple network events becomes dominant under a physically limited network bandwidth.

The overall performance measure of the proposed prefetching technique is the initial delay that is defined as the time period from the moment of the target click to the moment a video playing. The initial delay can be minimized by maximizing each design parameter. However, the design parameters are inter-related, requiring trade-offs and the careful choice of the proximity boundary, decision boundary and number of preparation modules.

### 4.2. Participants

User behavior analysis was performed with the data obtained from 10 participants to calibrate the proximity boundary and the decision boundary prior to the actual empirical tests. The actual experiments were conducted with the other 24 participants (16 males, eight females) with an average age of 26.0 (SD = 6.7). Among them, 10 participants were undergraduate students, and the others were students that graduated with an engineering background.

### 4.3. Procedures

All participants preliminarily took part in the calibration process for the gaze tracking with credit compensation of their class. After the calibration process, we debriefed them on the aim of the study and then instructed them to maintain a straight posture without excessive head movement for precise gaze tracking, but to freely click the listed videos and watch them just as in actual web video navigating circumstances. Even though the click number and the usage time were not restricted, the participants clicked the video 7 times (SD = 3.7) and used the website for about 22 min (SD = 5.4) on average for each trial. We examined three kinds of within-group empirical tests (*i.e.*, without-prefetching, cursor-based prefetching and the cursor-gaze prefetching method) to verify the actual and perceived initial delay according to the prefetching method. The first test (without-prefetching) was the control test; the second test employed the cursor-based prefetching technique; and the third test employed the proposed prefetching method based on the gaze-assisted intention prediction (cursor-gaze prefetching). All three tests were conducted in the same place as with the gaze tracker, even if the test was in the without-prefetching or cursor-based prefetching condition for the blind test. Each participant performed at least two trials for each test to secure more samples. After each trial, all participants were asked to answer the 5-point Likert scale questionnaire for the evaluation of the delay perception.

### 4.4. Test Environment

**Figure 7:**
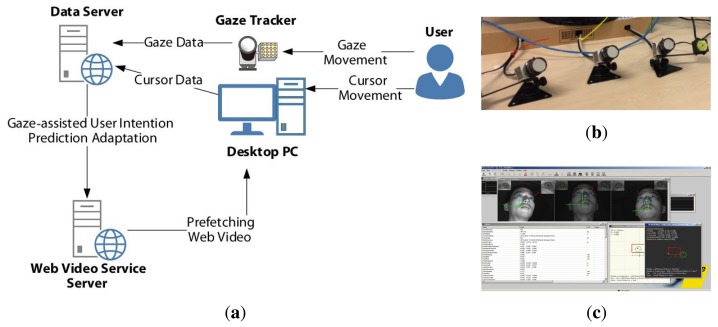
Test environment of the proposed gaze-assisted user intention prediction. (**a**) Framework of the implemented test system; (**b**) gaze trackers; (**c**) SmartEye Pro gaze tracking software.

Figure 7a shows the entire framework of the implemented test system, which includes a YouTube-like web video server, a data collecting server, a SmartEye Pro three-dimensional eye tracker (see Figure 7b) that has a 120-Hz sampling rate with real-time tracking software (see Figure 7c), a user desktop computer that is equipped with a 2.7-GHz processor, 8 GB of RAM and running Windows 8.1 64-bit and a 24-inch LCD monitor that has a resolution of 1920 × 1080 pixels. A Chrome browser extension for tracking the cursor movement by JavaScript and a medium software to transmit the gaze tracking data in real time though the UDP network from the desktop to the data collecting server were implemented. The data collecting server was based on Node.js and Redis to record all events, including the cursor and gaze coordinates, click event coordinates, page accesses, prefetching of candidates, prefetched video link ID, hit-ratio, preparation time, downlink bandwidth and initial delay along the timestamp.

Two versions of the decision module were implemented by JavaScript for comparison between the single modality (motor resource) and multimodality (motor and visual resource) using the Chrome web browser. A previously mentioned, our video website (see Figure 3a) was employed to apply our user intention prediction scheme. The cursor-based decision module used only the low-pass filtered cursor velocity as an input. The proposed cursor-gaze decision module used the low-pass filter cursor velocity and cursor-gaze distance as inputs. Each decision module was combined with the same previously mentioned candidate selection module to predict the user's intended targets.

We selected the most adjacent gaze event to the cursor event in the time domain to calculate the cursor-gaze distance, because the logging frequencies of the gaze and the cursor event were different. Then, the cursor velocity was obtained from the logged *x* − *y* coordinates and the timestamp. The test web page included various randomly rearranged video contents, such as news, music videos, technical reviews of consumer electronics and sports highlights.

## 5. Results

The design parameters and performance evaluation parameters obtained from 24 participants were analyzed to set the design guidelines for further research in user intention prediction for prefetching in web access. The relationship between design parameters and the performance evaluation can be adapted to other user intention prediction applications.

### 5.1. Hit-Ratio

**Figure 8:**
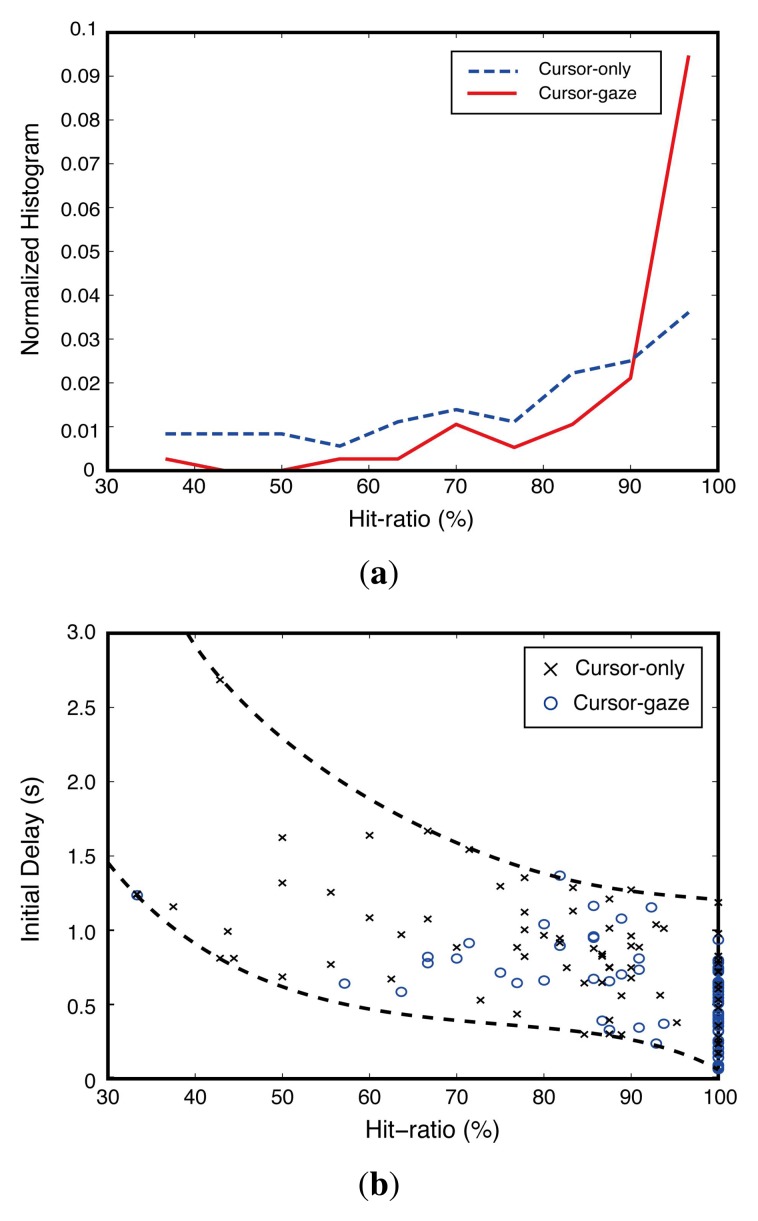
Relationship between hit-ratio and initial delay. (**a**) Normalized histogram of the hit-ratio; (**b**) influence of the hit-ratio.

The hit-ratio differences between the cursor-gaze case and cursor-only case were statistically significant (*F*(1,109) = 20.339, *p* < 0.0005) with the one-way ANOVA test. Figure 8a shows a normalized histogram of the hit-ratio obtained from the implemented test system. The cursor-gaze case shows a much higher histogram than the cursor-only case in an over 90% hit-ratio region. The cursor-gaze case performed more precise prefetching decisions than the cursor-only case. This means that the cursor-gaze interrelationship leads to a high confidence decision, preventing unnecessary prefetching. Figure 8b shows that the initial delay decreased as the hit-ratio increased. However, this initial delay improvement tends to be saturated when the hit-ratio exceeds about 85%. This means that the introduction of additional input devices for hit-ratio enhancement higher than 85% may not be necessary.

### 5.2. Preparation Time

**Figure 9:**
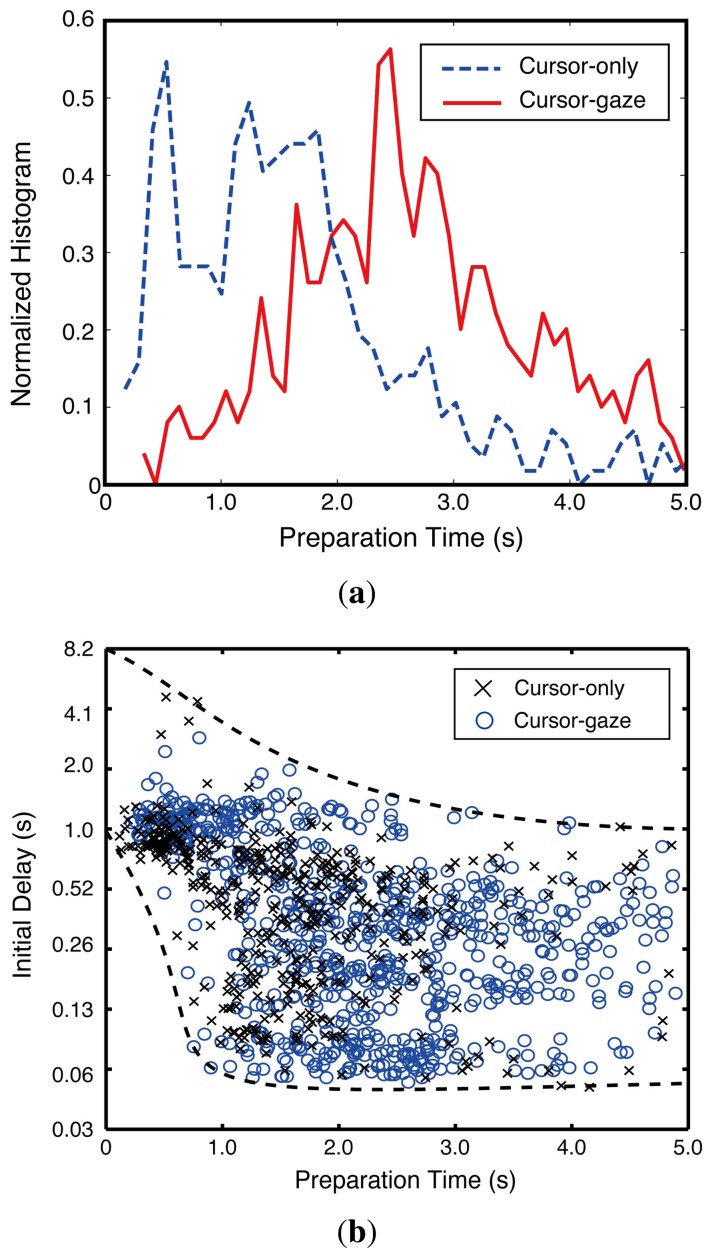
Influence of preparation time. (**a**) Normalized histogram of preparation time; (**b**) initial delay depending on preparation time (the vertical axis is drawn in binary logarithmic scale.

The preparation time differences between the cursor-gaze case and cursor-only case were statistically significant (*F*(1,970) = 75.121, *p* < 0.0005). Figure 9a illustrates the normalized histogram of the preparation time. The cursor-gaze case had a longer preparation time region than the cursor-only case. This distribution shift means that the cursor-gaze interrelationship allows activating the prefetching earlier than the cursor-only case. Figure 9b shows that the initial delay drastically decreased until the preparation time reached 1 s, although the gap between the upper bound and the lower bound of the initial delay tended to be saturated as the preparation time was higher than 3 s.

Since the web video start-up process requires player loading and initial video stream initiation, which take about 1 s, a drastic improvement in the initial delay was obtained when the preparation time was around 1–2 s. Once the preparation time exceeded the required time for the player and the initial video stream loading, an improvement in the initial delay beyond a 3-s preparation time was not significant. Hence, the preparation time securement of 2–3 s was the reasonable choice for the system design.

### 5.3. Number of Preparation Modules

**Figure 10:**
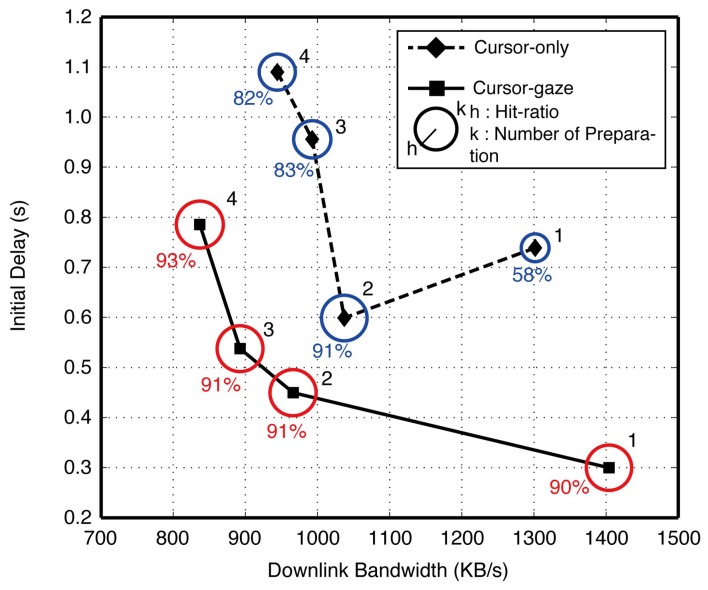
Initial delay and downlink bandwidth according to the number of preparation modules.

The number of preparation modules showed a statistically significant (*F*(3,107) = 8.820 and *p* < 0.0005) influence on the initial delay, while the influence on the hit-ratio was insignificant (*F*(3,107) = 1.705, *p* = 0.170) by the ANOVA test. Figure 10 shows the initial delay, hit-ratio and downlink bandwidth per preparation module with respect to the number of preparation modules. The downlink bandwidth decreased as the number of preparation modules increased, showing an ‘L’-shaped curve, because preparation modules shared limited total network bandwidth. Even though the initial delay decreased as the downlink bandwidth increased, the improvement was not significant when the downlink bandwidth exceeded a certain limit, because the server-client interaction time became a dominant portion of the initial delay. In the cursor-only case, despite the initial delay being improved as the number of preparation modules increased from one to two, an excess number of preparation modules degraded the initial delay. This means that an excessive number of preparation modules caused increased false decisions, which wasted network resources.

Meanwhile, the initial delay of the cursor-gaze case was smaller than the initial delay of the cursor-only case for all numbers of preparation modules due to the longer preparation time. Moreover, one preparation module showed the smallest initial delay for the cursor-gaze case, because the gaze modality assisted with securing a sufficiently high hit-ratio with one preparation module. Therefore, if a sufficiently high hit-ratio is secured, minimizing the number of preparation modules can maximize the initial delay reduction. From the results of the hit-ratio and preparation time, the impact on the initial delay of each parameter is verified as follows: the hit-ratio is deeply related to the first reduction step, which drastically drops the initial delay, and the preparation time has an impact on the additional initial delay reduction. A longer preparation time can be obtained by increasing the prefetching-on area in Figure 6 or by choosing a larger proximity boundary, *R*. However, these adjustments may degrade the hit-ratio and the initial delay after all.

### 5.4. Initial Delay

**Figure 11:**
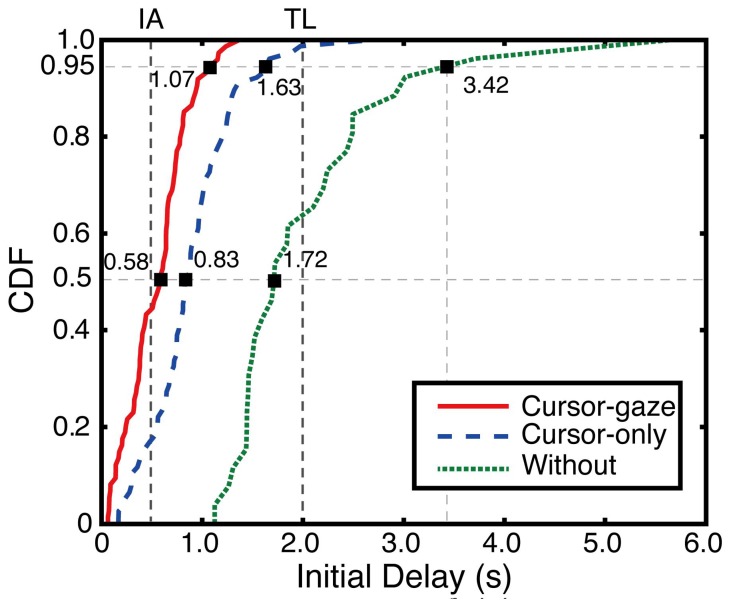
Measured CDF of the initial delay.

The initial delay differences between the without-prefetching case, cursor-gaze case and cursor-only case were statistically significant (*F*(2,139) = 59.617, *p* < 0.0005). Figure 11 shows a cumulative density function (CDF) of the initial delay for each version, demonstrating the overall performance of the proposed system. The cursor-gaze case recorded a 0.58-s initial delay as a median value, which was more than one-third of the without case that had a 1.72-s initial delay. Moreover, 45% of the cursor-gaze case was recorded under 0.5 s, which was perceived as an instantaneous access (IA), whereas only 18% of the cursor-only case and a negligible portion of the without case recorded under IA. On the other hand, all of the recorded initial delays of the cursor-gaze case were within about 1.5 s, which was much lower than the user tolerable limit (TL), *i.e.*, 2 s, while only 65% of the recorded initial delay falls within the TL in the without case.

### 5.5. User Perception

**Figure 12:**
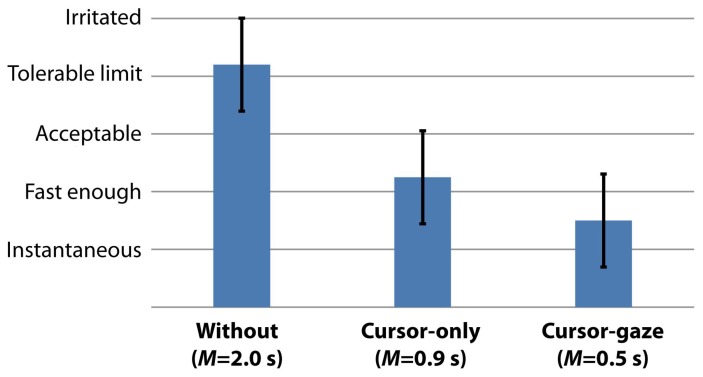
User perceived initial delay (lower is a faster response).

We evaluated the subjective user feedback with the five-point Likert scale questionnaire as a measure of the participants' perceived initial delay (from 1 = perceived as an instantaneous access, to 5 = perceived the delay as a irritation). The participants answered the questionnaire after the empirical test without informing about the type of prediction methods. Figure 12 shows that all of the participants perceived that the response of the cursor-gaze case was sufficiently fast (M = 1.5, SD = 0.7), whereas the response of the without case (M = 4.2, SD = 0.7) exceeds the tolerable limit. The perceived initial delay differences between the without prefetching case, cursor-gaze case and cursor-only case were statistically significant (*F*(2,69) = 69.701, *p* < 0.0005). The user feedback shows that the actual initial delay directly influences the the users' perception.

### 5.6. Summary

Table 2 shows a summary of the experimental results. The improvement of the mean value initial delay was 59% for the cursor-only case and 75% for the cursor-gaze case compared to the without case. The superior performance was obtained for the cursor-gaze case, because it had a higher hit-ratio (M = 91.6, SD = 13.8) and a longer preparation time (M = 2.4, SD = 0.65) than the cursor-only case.

**Figure 13:**
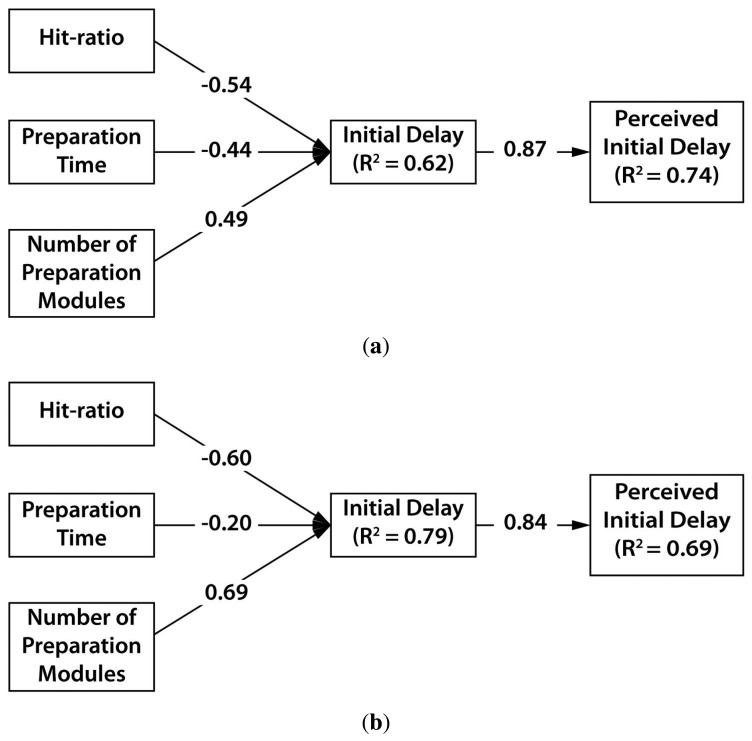
(**a**) Structural model of the cursor-only method; (**b**) structural model of the cursor-gaze method.

We validated how the hit-ratio, the preparation time and the number of preparation modules influenced the initial delay. Figure 13 shows structural models of the cursor-only and cursor-gaze case. Each path from one construct to other construct is the path loading, and *R*^2^ means the coefficient of determination. We firstly examined the path relationship between design parameters and initial delay in the cursor-only case. The initial delay in the cursor-only case showed statistically significant and negative influence from the hit-ratio (β = −0.54, *p* < 0.0005) and the preparation time (β = −0.44, *p* < 0.0005), as shown in Figure 13a. The number of preparation modules in the cursor-only case (β = 0.49, *p* < 0.0005) significantly and positively influenced the initial delay. The three design parameters were found to be significant predictors (*R*^2^ = 0.62, *F*(3,50) = 30.250, *p* < 0.0005) of the initial delay. The initial delay in the cursor-gaze case showed statistically significant and negative influence from the hit-ratio (β = −0.602, *p* < 0.0005) and preparation time (β = −0.204, *p* < 0.005), as shown in Figure 13b. The number of preparation modules in the cursor-gaze case (β = 0.688, *p* < 0.0005) significantly and positively influenced the initial delay. The structural model of the cursor-gaze case (*R*^2^ = 0.79, *F*(3,53) = 69.050, *p* < 0.0005) can statistically explain more than the model of the cursor-only case. The perceived initial delay is significantly influenced by the actual initial delay (β = 0.87, *p* < 0.0005), which is similar to the cursor-gaze case (β = 0.84, *p* < 0.0005). The initial delay was found to be a good predictor of the user perception for the cursor-gaze case (*R*^2^ = 0.69, *F*(1,23) = 52.381, *p* < 0.0005) and the cursor-only case (*R*^2^ = 0.74, *F*(1,23) = 67.375, *p* < 0.0005).

## 6. Conclusions

This paper proposed a gaze-assisted user intention prediction technique based on the threaded interaction model, which can take into account the sequential and concurrent flow of the threads in the human mind, behavior, as well as the device and service aspect. The TIM suggests the necessity of a separate candidate selection module, decision module and preparation module utilizing at least two different types of sensors to achieve a high hit-ratio and a long preparation time at the same time. The proposed scheme was applied to a web video prefetching application. The experimental results demonstrated significant reduction of initial delay and UX improvement, proving the effectiveness of the proposed TIM.

## Figures and Tables

**Table 1:** Summarized horizontal event distribution.

	**Blank**	**Thumbnail**	**Title**	**Blank**	**Video Player**	**Blank**

**Horizontal Coordinate**	**0–480px**	**480–545px**	**545–785px**	**785–810px**	**810–1450px**	**1450–1920px**
Gaze Event	0.03%	0.22%	0.13%	4.02%	91.7%	3.91%
Cursor Event	65.3%	8.29%	17.9%	3.98%	4.14%	0.29%
Click Event	0.00%	11.86%	87.11%	1.03%	0.00%	0.00%

**Table 2:** Summarized performance of the proposed user intention prediction methods.

**Parameters**	**Hit-Ratio (%)**			**Preparation Time (ms)**			**Initial Delay (ms)**		

**Values**	**M**	**SD**	**SE**	**M**	**SD**	**SE**	**M**	**SD**	**SE**
Without	-	-	-	-	-	-	2062.13	959.94	188.26
Cursor-only	76.93	20.14	2.74	1507.67	738.81	84.20	875.00	444.75	60.52
Cursor-gaze	91.67	13.88	1.84	2443.86	653.72	86.59	490.89	313.14	41.48
